# Anti-Inflammatory Effect of Titrated Extract of *Centella asiatica* in Phthalic Anhydride-Induced Allergic Dermatitis Animal Model

**DOI:** 10.3390/ijms18040738

**Published:** 2017-03-30

**Authors:** Ju Ho Park, Ji Yeon Choi, Dong Ju Son, Eun Kyung Park, Min Jong Song, Mats Hellström, Jin Tae Hong

**Affiliations:** 1 College of Pharmacy and Medical Research Center, Chungbuk National University, 194-31 Osongsaengmyeong 1-ro, Osong-eup, Heungduk-gu, Cheongju 361-951, Korea; jhp31888@naver.com (J.H.P.); cjy8316@hanmail.net (J.Y.C.); sondj1@chungbuk.ac.kr (D.J.S.); 2 Department of Obstetrics & Gynecology, Daejeon St. Mary’s Hospital, The Catholic University of Korea, 64 Daeheung-Ro (Daeheung-dong), Jung-gu, Daejeon 301-723, Korea; guevara614@catholic.ac.kr (E.K.P.); bitsugar@catholic.ac.kr (M.J.S.); 3 Laboratory for Transplantation and Regenerative Medicine, Sahlgrenska Academy, University of Gothenburg, Gothenburg 411-15, Sweden; mats.hellstrom@gu.se

**Keywords:** titrated extract of *Centella asiatica*, skin inflammation, atopic dermatitis, NF-κB, cytokine, IgE

## Abstract

*Centella asiatica* has potent antioxidant and anti-inflammatory properties. However, its anti-dermatitic effect has not yet been reported. In this study, we investigated the anti-dermatitic effects of titrated extract of *Centella asiatica* (TECA) in a phthalic anhydride (PA)-induced atopic dermatitis (AD) animal model as well as in vitro model. An AD-like lesion was induced by the topical application of five percent PA to the dorsal skin or ear of Hos:HR-1 mouse. After AD induction, 100 μL of 0.2% and 0.4% of TECA (40 μg or 80 μg/cm^2^) was spread on the dorsum of the ear or back skin three times a week for four weeks. We evaluated dermatitis severity, histopathological changes and changes in protein expression by Western blotting for inducible nitric oxide synthase (iNOS), cyclooxygenase-2 (COX-2), and NF-κB activity, which were determined by electromobility shift assay (EMSA). We also measured TNF-α, IL-1β, IL-6, and IgE concentration in the blood of AD mice by enzyme-linked immunosorbent assay (ELISA). TECA treatment attenuated the development of PA-induced atopic dermatitis. Histological analysis showed that TECA inhibited hyperkeratosis, mast cells and infiltration of inflammatory cells. TECA treatment inhibited expression of iNOS and COX-2, and NF-κB activity as well as the release of TNF-α, IL-1β, IL-6, and IgE. In addition, TECA (1, 2, 5 μg/mL) potently inhibited Lipopolysaccharide (LPS) (1 μg/mL)-induced NO production, expression of iNOS and COX-2, and NF-κB DNA binding activities in RAW264.7 macrophage cells. Our data demonstrated that TECA could be a promising agent for AD by inhibition of NF-κB signaling.

## 1. Introduction

Atopic dermatitis (AD) is a common chronic inflammatory skin disease inducing intense itching, edema, erythema, thickening, severe pruritus, and eczematous lesions of the skin. Several genetic and environmental factors and immune responses are implicated for the development of AD [1]. Elevated production of serum IgE against many kinds of inhaled allergens and secretion of T helper (Th) 2 cytokines are the main causes of AD [2,3]. Mast cell activation mediated by IgE leads to a release of various chemical mediators which results in infiltration of inflammatory cells such as eosinophils and lymphocytes into the skin lesion [4]. CD4^+^ T cells and mast cells in the skin lesions are also involved in the pathogenesis of AD [5,6,7,8]. It has been reported that 2,4-dinitrochlorobenzene (DNCB)-induced AD-like skin lesion mouse model showed increased serum IgE and Th2 cytokines such as IL-4, IL-5, and IL-13 [9,10]. These cytokines have direct effects on epidermal keratinocytes, which produce pro-inflammatory cytokines that induce infiltration of immune cells into inflammatory skin lesions [11]. These data indicate that inflammation and activation of immune cells could be significant for the development of AD.

Nuclear factor-κB (NF-κB) is an important transcription factor associated with the allergic inflammatory response in AD. Many studies have shown that NF-κB is an important factor in the regulation of various immune responses in allergic disorders such as AD, asthma, and rheumatoid arthritis [12,13,14]. Since the activation of NF-κB may exacerbate the allergic inflammation by enhancing the production of inflammatory cytokines and chemokines, various methods have been developed to inhibit NF-κB activation. Moreover, NF-κB inhibitor, IMD-0354 inhibited abnormal proliferation of mast cells, and reduced the allergic response [15]. Potent immunosuppressive drugs such as tacrolimus, corticosteroids, and cyclosporine have been studied as therapeutic agents for AD through inhibition of cytokine production [16]. However, these agents cause severe reverse effects such as tachyphylaxis, recurrence, and exacerbation of AD [17,18]. Thus, other new drugs showing no side effects with strong pharmacological properties could be developed.

*Centella asiatica*, known by the common name Gotu kola, is a traditional herbal medicine that has been used to exert pharmacological effects in dermatology [19]. The *Centella asiatica* herb is used in the treatment of skin lesions such as burn wounds, excoriations, or eczema as well as in non-dermatological diseases such as diabetic complications [20], and neurodegenerative disorders [21]. *Centella asiatica* has also been effective in chronic venous insufficiency by improvement of microcirculation [22]. The *Centella asiatica* extract was registered in International Nomenclature of Cosmetic Ingredients (INCI) as an ingredient of cosmetics [23]. Although various pharmacological effects of *Centella asiatica* have been reported, its anti-dermatitic effect has not yet been reported. Therefore, we investigated the anti-dermatitic effects of titrated extract of *Centella asiatica* and action mechanism in a phthalic anhydride-induced atopic dermatitis animal model as well as in vitro model.

## 2. Results

### 2.1. Effects of TECA Treatment on Ear Thickness and Morphology

**Figure 1:**
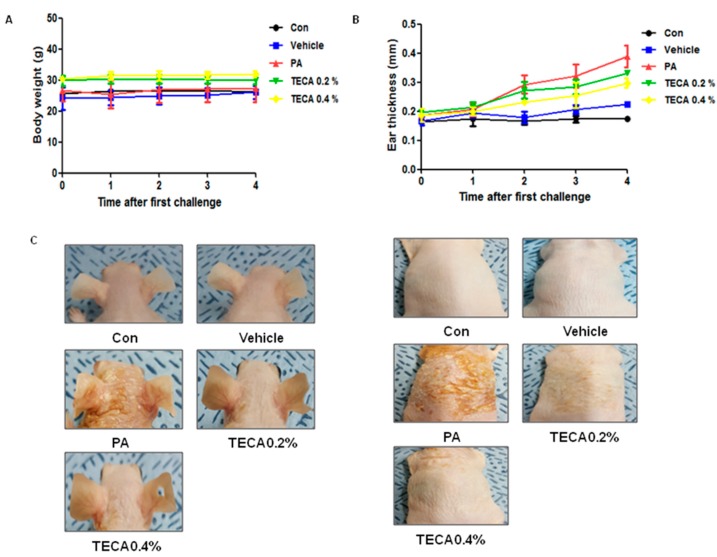
Differences in body weight, ear thickness, ear phenotypes, and back phenotypes. Phthalic anhydride (PA) solution was repeatedly applied to the dorsum of ear and back three times a week during topical application of Titrated extract of Centella asiatica (TECA). After four weeks, body weight (**A**) and ear thickness (**B**) were observed at least three times by following the procedure described in Materials and Methods. Phenotypes (**C**) were randomly selected by one mouse/group. Data shown are the mean ± SD (*n* = 10).

Changes in body weight were measured during the experimental period. No significant difference in body weight was detected after any of the treatments (Figure 1A). To investigate whether or not treatment with TECA can suppress the changes in ear phenotype induced by PA treatment, ear thickness and morphology of ear were observed. Ear thickness rapidly increased in PA treated mice compared to control or vehicle treated mice. On the other hand, ear thickness in TECA treated mice was slowly increased in a dose-dependent manner (Figure 1B). Furthermore, symptoms consisting of erythma, edema, and erosion were observed in the PA treated group compared with the control or vehicle treated group. These changes of ear and back morphology and ear thickness were dramatically reversed upon TECA treatment (Figure 1C).

### 2.2. Effect of TECA Treatment on Lymph Node Weight and IgE Concentration as Well as on Expression of iNOS and COX-2

**Figure 2:**
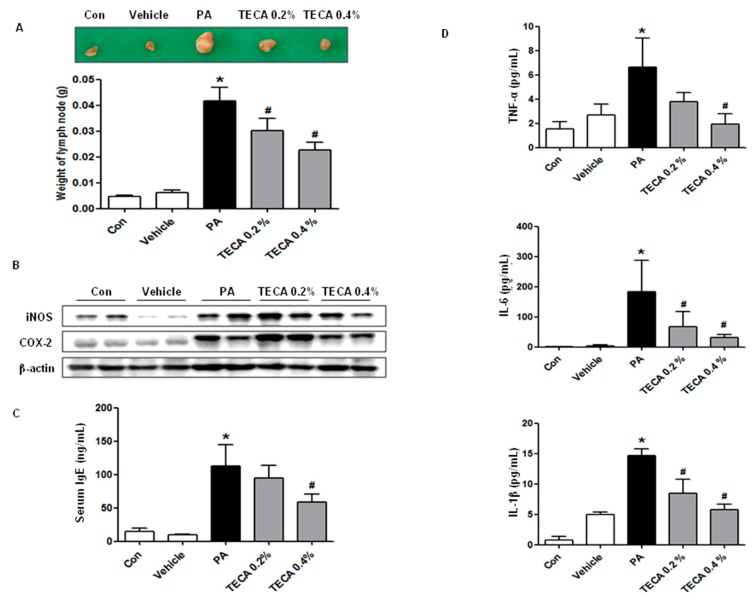
Changes in auricular lymph node weight, expression level of iNOS and COX-2 protein in lymph node, and serum cytokine concentration. After final treatment, mice from each group were sacrificed under anesthesia. The auricular lymph nodes were then harvested from the neck regions of the mice using a microscissor, after which they were weighed (**A**); Alteration of the expression of the two proteins was measured by Western blotting (**B**); Serum used to measure the cytokine concentration was prepared from blood samples collected from the abdominal veins of mice. Serum IgE (**C**), TNF-α, IL-6, and IL-1β (**D**) concentration were quantified by enzyme-linked immunosorbent assay (ELISA). Data shown are gained from the same mice treated shown in Figure 1. Data shown are the mean ± SD (*n* = 10). * *p* < 0.05 is the significance level compared to the control group. ^#^ *p* < 0.05 is the significance level compared to the PA treated group.

We investigated whether or not TECA could suppress the increases in lymph node weight and IgE concentration. To accomplish this, we evaluated the auricular lymph node weight and serum IgE concentration. PA treatment induced an increase in lymph node weight compared with control or vehicle treated mice. However, the weight of lymph node was significantly reduced in the TECA treated mice (Figure 2A). In addition, protein expressions of iNOS and COX-2 were significantly upregulated in PA treated AD mice, but significantly suppressed by TECA 0.4% treatment (Figure 2B). It is well known that hyperproduction of IgE is one of the characteristic features of allergic hypersensitivity as well as an indicator of the magnitude of the allergic immune responses in the development of AD [24]. The serum IgE concentration was measured in the blood of mice to determine whether TECA suppressed the allergic responses induced by PA treatment. Repeated topical application of PA solution induced a significant increase in serum IgE concentration. However, a significant decrease of IgE concentration was observed in the TECA treated group (Figure 2C).

### 2.3. Effect of TECA Treatment on the Release of Inflammatory Cytokines

To determine if TECA treatment could induce alterations in the inflammatory cytokines release in PA-induced skin inflammation, the level of TNF-α, IL-6, and IL-1β was measured in mouse serum of control, vehicle, PA and PA + TECA treated group. The level of TNF-α, IL-6, and IL-1β was generally higher in the PA treated group than the control or vehicle treated group. However, these levels in the TECA treated group were dramatically decreased to the level of the control or vehicle treated group (Figure 2D).

### 2.4. Effect of TECA Treatment on Inflammatory Responses in Ear and Back

**Figure 3:**
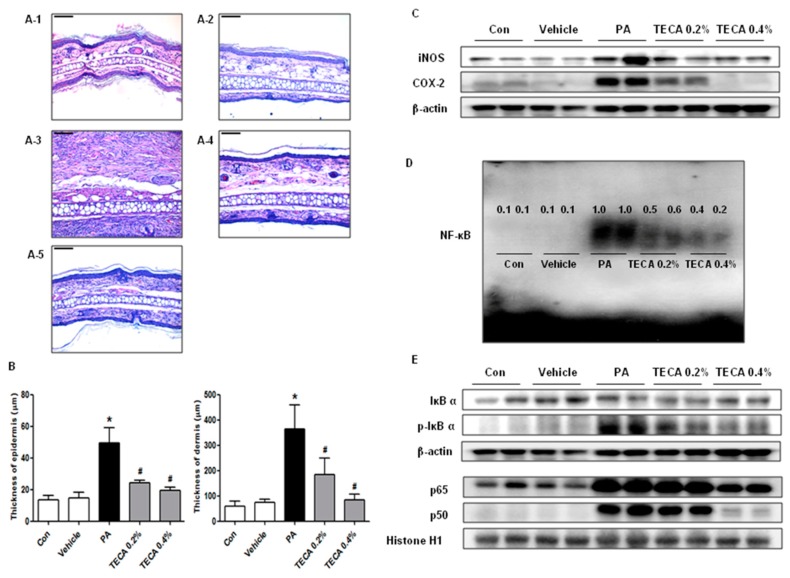
Histopathological analysis of ear tissue and the inhibitions of NF-κB DNA binding activity by topical application of TECA in ear skin. Histopathology of ear skin in control (**A-1**), vehicle (**A-2**), PA (**A-3**), PA + TECA 0.2% (**A-4**), and PA + TECA 0.4% (**A-5**). PA solution was repeatedly applied to the dorsum of ears during topical application of TECA. Histopathological changes in the slide sections of ear tissue were identified by staining with hematoxylin and eosin followed by observation at 200× magnification (Scale bars, 100 μm). (**A**) Histological images and (**B**) thickness of the epidermis and dermis. Alteration of the expression of iNOS and COX-2 proteins were measured by Western blotting (**C**); (**D**) Effect of TECA on NF-κB DNA binding activity in ear skin. The activation of NF-κB was investigated using electromobility shift assay (EMSA) as described in Materials and Methods. Nuclear extracts from homogenized ear skin tissue were incubated in binding reactions of ^32^P-end-labeled oligonucleotide containing the NF-κB sequence (numbers: relative expression). (**E**) Effect of TECA on translocation of the subunits of NF-κB (p50 and p65) into nucleus, and phosphorylation of IκBα in cytosol in ear skin. Equal amounts of nuclear proteins (20 μg/lane) or total proteins (20 μg/lane) were subjected to 10% SDS-PAGE, and expression of p50, p65, IκBα, and p-IκBα protein were detected by Western blotting using specific antibodies. Histone h1 protein and β-actin protein were used here as an internal control. Data shown are gained from the same mice treated shown in Figure 1. Data shown are the mean ± SD (*n* = 10). * *p* < 0.05 is the significance level compared to the control group. ^#^ *p* < 0.05 is the significance level compared to the PA treated group.

**Figure 4:**
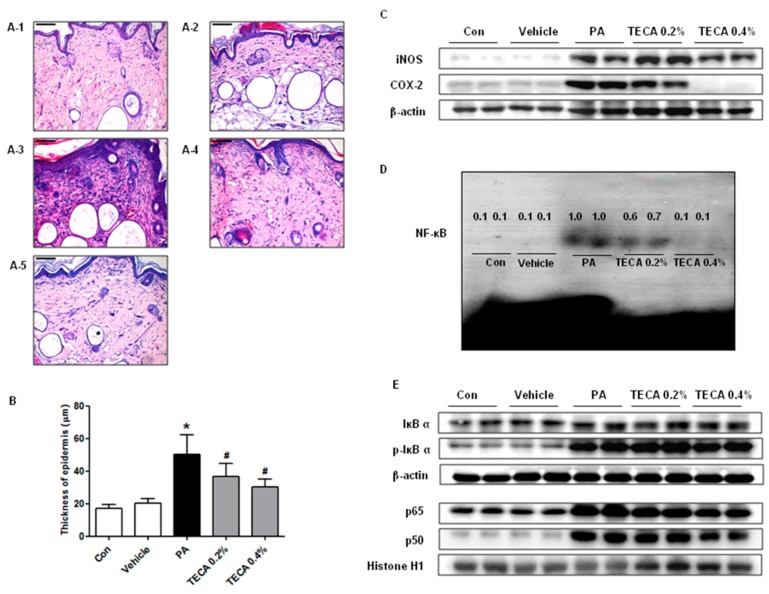
Histopathological analysis of back tissue and the inhibitions of NF-κB DNA binding activity by topical application of TECA in back skin. Histopathology of back skin in control (**A-1**), vehicle (**A-2**), PA (**A-3**), PA + TECA 0.2% (**A-4**), and PA + TECA 0.4% (**A-5**). PA solution was repeatedly applied to the back skin during topical application of TECA. Histopathological changes in the slide sections of back tissue were identified by staining with hematoxylin and eosin followed by observation at 200× magnification (Scale bars, 100 μm). (**A**) Histological images and (**B**) thickness of the epidermis. Alteration of the expression of iNOS and COX-2 proteins were measured by Western blotting (**C**); (**D**) Effect of TECA on NF-κB DNA binding activity in back skin. The activation of NF-κB was investigated using EMSA as described in Materials and Methods. Nuclear extracts from homogenized back skin tissue were incubated in binding reactions of ^32^P-end-labeled oligonucleotide containing the NF-κB sequence (numbers: relative expression); (**E**) Effect of TECA on translocation of the subunits of NF-κB (p50 and p65) into nucleus, and phosphorylation of IκBα in cytosol in back skin. Equal amounts of nuclear proteins (20 μg/lane) or total proteins (20 μg/lane) were subjected to 10% SDS-PAGE, and expression of p50, p65, IκBα, and p-IκBα protein were detected by Western blotting using specific antibodies. Histone h1 protein and β-actin protein were used here as an internal control. Data shown are gained from the same mice treated shown in Figure 1. Data shown are the mean ± SD (*n* = 10). * *p* < 0.05 is the significance level compared to the control group. ^#^ *p* < 0.05 is the significance level compared to the PA treated group.

To investigate the suppressive effect of TECA treatment on ear and back histology, histological analysis of the ear and back skin were performed (Figure 3A and Figure 4A). The epidermis and dermis of the ear (Figure 3B), and the epidermis of the back (Figure 4B) were thicker in PA treated group than in the control or vehicle treated group. However, the thickness of them was greatly decreased in the TECA treated group in a dose-dependent manner. In addition, protein expressions of iNOS and COX-2 were significantly upregulated in PA treated AD mice, but significantly suppressed by TECA 0.4% treatment (Figure 3C and Figure 4C).

### 2.5. Effect of TECA on NF-κB DNA Binding Activity in PA-Induced AD Mice

NF-κB is implicated for inflammatory responses in AD model. To investigate whether TECA can inhibit NF-κB activation in PA-induced AD model, nuclear extracts from ear and back skin tissue were prepared and assayed with NF-κB DNA binding by EMSA. PA treated mice showed significant NF-κB binding activity when compared to control group in both ear and back skin. On the contrary, NF-κB binding activity in TECA treated mice was significantly inhibited when compared with PA treated mice (Figure 3D and Figure 4D). In addition, as shown in Figure 3E and Figure 4E, PA treated mice showed significant IκBα degradation in cytosolic fraction when compared to the control group in both ear and back skin. On the contrary, IκBα degradation in TECA treated mice was reduced significantly when compared with PA treated mice. PA treated mice also showed increase in the relocalization of p65 and p50 in nucleus. In contrast, TECA inhibited translocation of p65 and p50 into the nuclear in a dose-dependent manner (Figure 3E and Figure 4E).

### 2.6. Effect of TECA on LPS-Induced NO Production, and iNOS and COX-2 Expression in RAW264.7 Cells

**Figure 5:**
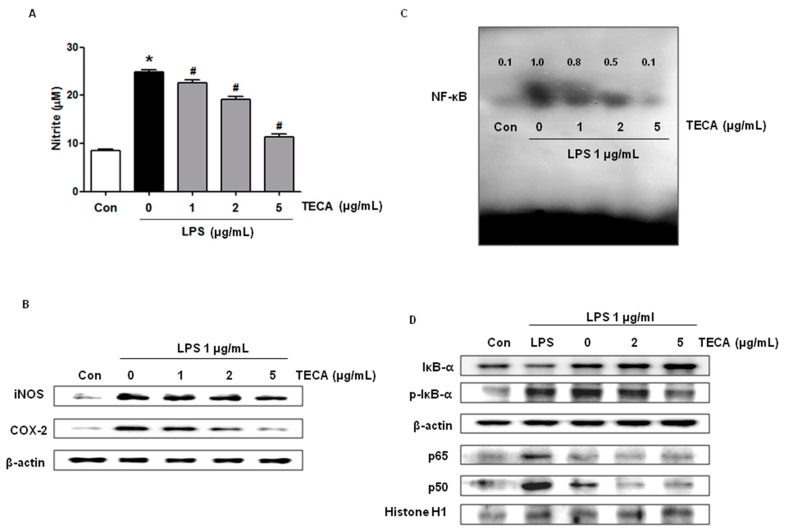
Effects of TECA on LPS-induced NO production, and iNOS and COX-2 expression in RAW264.7 cells. RAW 264.7 cells were pre-treated with different concentration (1, 2, and 5 μg/mL) of TECA for 2 h and then stimulated with LPS (1 μg/mL) for 24 h. Effect of TECA on LPS-induced NO production was measured by the Griess reaction as described in Materials and Methods (**A**); The expression of iNOS and COX-2 after stimulated 24 h was determined by Western blot (**B**); Effect of TECA on LPS-indcued NF-κB DNA binding activity was measured by EMSA as described in Materials and Methods (**C**); Effects of TECA on LPS-induced phosphorylation of IκBα in cytosol, and translocation of the subunits of NF-κB (p50 and p65) into nucleus were measured by Western blot (**D**). Data shown are gained from the same mice treated shown in Figure 1. Data shown are the mean ± SD (*n* = 10). * *p* < 0.05 is the significance level compared to the control group. ^#^ *p* < 0.05 is the significance level compared to the PA treated group.

The effect of TECA on LPS-induced NO production in RAW264.7 cells was investigated by measuring the released nitrite in the culture medium by Griess reaction. After co-treatment with LPS and TECA (1, 2, 5 μg/mL) for 24 h, LPS-induced elevation of nitrite concentration in the medium were decreased in a concentration-dependent manner (Figure 5A). In addition, we determined iNOS and COX-2 expression by Western blot analysis. As shown in Figure 5B, LPS-induced iNOS and COX-2 expression were significantly inhibited by TECA (1, 2, 5 μg/mL) in a concentration-dependent manner.

### 2.7. Effect of TECA on NF-κB DNA Binding Activity in RAW 264.7 Cells

Because activation of NF-κB is critical for induction of both iNOS and COX-2 by LPS or other inflammatory cytokines, we determined whether TECA might suppress NF-κB activation in LPS-activated RAW264.7 cells. RAW264.7 cells were co-treated with LPS and TECA for 1 h, respectively, which is the time to activate NF-κB maximally from its LPS treatment (data are not shown). Nuclear extracts from co-treated cells were prepared and assayed NF-κB DNA binding by EMSA. In RAW264.7 cells, LPS induced a strong NF-κB binding activity, which was markedly inhibited by co-treatment with TECA in a concentration-dependent manner (Figure 5C). We further investigated the inhibitory effect of TECA on the translocation of NF-κB subunit and IκB phosphorylation. Consistent with the inhibitory effect on NF-κB activity, nuclear translocation of p65 and p50 was inhibited in a concentration-dependent manner, and the LPS-induced phosphorylation of IκBα was also inhibited by TECA in a concentration-dependent manner (Figure 5D).

## 3. Discussion

Topical application of corticosteroids have been used for the treatment of AD because of their great anti-inflammatory and anti-allergic activities [25]. However, they cause irreversible side effects from long-term usage such as common pathogenic infections and immune suppression [26]. For this reason, usage of natural products including various plants, herbs, flowers, yeasts, and fungi is being emphasized as anti-inflammatory and anti-allergic agents. AD is characterized by skin inflammation with eczema-like lesions, itching, and dry skin [27]. In an experimental model, the thickness of ear, epidermis and dermis were important indexes to evaluate the severity of skin inflammation. We found that TECA effectively reduces the skin inflammation and allergic responses induced by PA treatment. In in vitro assay, we also found that TECA inhibited LPS-induced inflammatory responses. It was proven that *Centella asiatica* has an excellent effect on deposition of extracellular matrix proteins. It stimulates proliferation of fibroblasts, increases the synthesis of collagen, decreases metalloproteinases activity and thus increases the deposition of collagen and intracellular free proline levels [28,29,30,31]. It also inhibits the inflammatory phase of wound healing [32]. TECA contains asiatic acid (30%), madecassic acid (29–30%), and asiaticoside (40%). The influence of asiatic acid, madecassic acid, and asiaticoside on human skin fibroblast type I collagen synthesis was also found [33]. In a recent study, it was reported that components of *Centella asiatica*, asiaticoside and madecassoside possess wound healing, collagen synthesis, as well as vasodilation activities [34]. These effects are associated with the reduced activation of macrophages and the production of IL-1β [35]. Furthermore, it has been reported that the component of *Centella asiatica*, madecassic acid, plays a role in anti-inflammatory activity through the downregulation of iNOS and COX-2 expression and TNF-α, IL-1β, and IL-6 release in RAW264.7 macrophage cells [36]. *Centella asiatica* applied in the recommended doses is not toxic and possible side effects are rare [19]. These data thus indicate that TECA could be applicable for AD.

It is well known that macrophages play an important role in both acquired and nonspecific immune responses. Activation of macrophage leads to various series of responses including the production of pro-inflammatory cytokines which exert their inflammatory effects by activating a diverse spectrum of signaling cascades in the cells that lead to the induction of inflammatory genes such as iNOS and COX-2 [37]. In this study, PA-induced expression of iNOS and COX-2 was also reduced by TECA in the skin as well as cultured macrophage. IgE-induced activation of mast cells, which resulted in the release of various allergic mediators such as cytokines and histamine [38]. Therefore, the low level of IgE induces lesser allergic responses, and reduces the levels of cytokine. In this regard, TECA potently reduced the level of IgE and release of inflammatory cytokine. These data indicate that TECA could inactivate macrophage in the skin, thus lead to less skin inflammation and atopic responses.

NF-κB is implicated for cytokine release, which is important for anti-inflammatory activity. Pro-inflammatory cytokines, including IL-4, IL-6, IL-1β, and TNF-α, commonly contribute to the regulation of inflammation and immune responses in AD skin lesion [39]. Release of IL-4 primarily regulates hyper-production of IgE [40], and expression of TNF-α and IL-6 stimulate the synthesis of acute phase response protein, which attenuates secretion of IgE and disruption of skin barrier function during allergic reactions [41,42]. In a recent study, *Spirodela polyrhiza* remarkably inhibited expression levels of NF-κB and p-IκBα as well as inflammatory cytokines such as IL-4, IL-6, and TNF-α in AD mice model [43]. Tanaka et al. demonstrated topical application of IMD-0354, an NF-κB inhibitor, is effective in suppressing the activation of NF-κB and in reducing the development of AD in atopic NC/NgaTnd mice [44]. Moreover, it was reported that treatment of NF-κB inhibitor *Xanthii fructus* (XF) strongly suppressed IL-4, IL-1β, IFN-γ and TNF-α in AD-like skin lesions [45]. In addition, several natural products inhibited AD development through inhibition of cytokine releases. In TPA-induced skin inflammation, TNF-α and IL-1β in the serum were reduced by 70% ethanol extract from *Asparagus cochinchinensis* [46]. Following treatment with *Liriope platyphylla* (LP) extract, expression of IL-6 and VEGF was significantly reduced in ear tissue of IL-4/Luc/CNS-1 Tg mice treated with PA [5]. In the present study, the levels of two cytokines (TNF-α and IL-6) were elevated in the serum of mice treated with PA, but significantly reduced cytokine release was observed in the TECA treated group. In both PA-induced atopic dermatitis animal model and RAW 264.7 murine macrophage cells, TECA also decreased the degradation of IκBα and nuclear translocation of NF-κB. It has been reported that asiaticoside, a component of *Centella asiatica*, plays a role in the anti-inflammatory effect via downregulation of NF-κB signaling pathway [47]. In our present study, the data demonstrated that TECA attenuates activation of NF-κB, contributing to the reduced TNF-α, IL-6, and IL-1β level and expression of iNOS and COX-2. Therefore, our data suggest that TECA should be considered a candidate agent for AD.

## 4. Materials and Methods

### 4.1. Ethical Approval

The experimental protocols were carried out according to the guidelines for animal experiments of the Institutional Animal Care and Use Committee (IACUC) of the Laboratory Animal Research Center at Chungbuk National University, Korea (CBNUA-929-16-01). All efforts were made to minimize animal suffering, and to reduce the number of animals used. All mice were housed in three mice per cage with an automatic temperature control (21–25 °C), relative humidity (45–65%), and 12 h light–dark cycle illuminating from 08:00 a.m. to 08:00 p.m. Food and water were available ad libitum. They were fed a pellet diet consisting of crude protein 20.5%, crude fat 3.5%, crude fiber 8.0%, crude ash 8.0%, calcium 0.5%, and phosphorus 0.5% per 100 g of the diet (collected from Daehan Biolink, Chungcheongbuk-do, Korea). During this study, all mice were particularly observed for normal body posture, piloerection, ataxia, urination, etc., 2 times per day.

### 4.2. Preparation and Extraction of Centella asiatica

Collected aerial parts of *Centella asiatica* were oven-dried at 50 °C and then powdered using a milling machine. The powdered plant (1 kg) was extracted with 75% (*v*/*v*) ethanol (3 × 4 L, 3 days each) at room temperature. The extracts were filtrated with a depth-filter coated with active carbon and concentrated at 80 °C under reduced pressure. The concentrate was divided to precipitate and filtrate fraction by filtration. The precipitate fraction was dried at 50 °C to make asiaticoside powder; its yield was 0.12% (*w*/*w*) of dried plant. Subsequently, the filtrates fraction was hydrolyzed with an alkaline solution containing sodium hydroxide (1% *w*/*v*) at 80 °C. It was then concentrated, precipitated and dried using the procedure described above. The yield of the genins (asiatic acid and madecassic acid) powder obtained from the filtrate fraction was 0.18% (*w*/*w*) of dried plant. Both powder extracts were mixed to give the titrated extract of *Centella asiatica*. The components of the titrated extract of *Centella asiatica* were asiaticoside (40%), asiatic acid (30%), and madecassic acid (29–30%) (Table 1). All solvents used were of commercial grade and obtained from Dongkook Pharmaceutical Company, Chungbuk, Korea.

### 4.3. Animal Treatment

The protocols for the animal experiment used in this study were carefully reviewed for ethical and scientific care procedures and approved by the Chungbuk National University-Institutional Animal Care and Use Committee (Approval Number CBNUA-929-16-01). Hos:HR-1 mice (eight-week-old, *n* = 40) were randomly divided into one of four groups. In the first group (Vehicle, *n* = 10), 100 μL of AOO (4:1 acetone: olive oil, *v*/*v*: AOO) was spread on the dorsum of the ears and back skin three times a week for four weeks. In the second group (phthalic anhydride (PA), *n* = 10), 100 μL (20 μL/cm^2^) of 5% phthalic anhydride solution was applied. The third group (TECA 0.2%, *n* = 10) and fourth group (TECA 0.4%, *n* = 10) were applied with PA, and 3 h later 100 μL of 0.2% and 0.4% titrated extract of *Centella asiatica* (40 μg or 80 μg/cm^2^) were applied. Age-matched Hos:HR-1 mice were used as the control group (Control, *n* = 10).

### 4.4. Measurement of Ear Thickness, and Body and Lymph Node Weight

Body weights of all mice were measured during the experimental period using an electronic balance (Mettler Toledo, Greifensee, Switzerland) once a week for 4 weeks. Additionally, weights of lymph nodes were measured using an electronic balance lymph nodes were collected from sacrificed mice and weighed using an electronic balance (Mettler Toledo, Greifensee, Switzerland). Thickness of ear skin was measured using a thickness gauge (Digimatic Indicator, Matusutoyo Co., Tokyo, Japan).

### 4.5. Histological Techniques

The ear and back skins were removed from mice, fixed with 10% formalin, embedded in paraffin wax, routinely processed, and then sectioned into 5 μm thick slices. The skin sections were then stained with hematoxylin and eosin (H & E). The thickness of the epidermis and dermis were also measured using the Leica Application Suite (Leica Microsystems, Wetzlar, Germany).

### 4.6. Mesurement of Serum IgE Concentration

IgE level in the serum was measured by enzyme-linked immunosorbent assay (ELISA) using the mouse IgE kit (Shibayagi, Inc., Gunma, Japan), according to the manufacturer’s instructions. The final concentration of IgE was calculated using a linear regression equation obtained from standard absorbance values.

### 4.7. Cytokine Assay

By the end of the study period, blood specimens were collected. Serum levels of mouse TNF-α, IL-6, and IL-1β were measured by enzyme-linked immunosorbent assay (ELISA) kits provided by Thermoscientific Inc. (Meridian Rd, Rockford, IL, USA) according to the manufacturer’s protocol.

### 4.8. Western Blot Analysis

One hundred milligrams of skin or ear tissues or about 1 × 10^6^ cells were harvested and homogenized with a lysis buffer (50 mM Tris pH 8.0, 150 mM NaCl, 0.2% Sodium dodecyl sulfate (SDS), 1 mM phenyl methylsulfonyl fluoride (PMSF), and 0.5% sodium deoxycholate). After lysis, the lysates were centrifuged at 13,000 rpm for 20 min. Equal amounts of protein (20 μg) were denatured at 95 °C for 5 min after mixing with 5 μL of SDS loading buffer were applied on SDS/10% polyacrylamide gel for electrophoresis and were transferred to nitrocellulose membranes (Hybond ECL, Amersham Pharmacia Biotech Inc., Piscataway, NJ, USA). The membrane was incubated for 4 h at room temperature with specific antibodies: rabbit polyclonal antibodies against iNOS, COX-2, p65 and IκB-α (1:500), and mouse monoclonal antibody against p50 (1:500) (Santa Cruz Biotechnology Inc., Santa Cruz, CA, USA) were used in study. The blot was then incubated with the corresponding conjugated anti-rabbit immunoglobulin G-horseradish peroxidase (Santa Cruz Biotechnology Inc., Santa Cruz, CA, USA). Band signals were detected on X-ray film using enhanced chemiluminescence (ECL) detection reagents.

### 4.9. Gel Electromobility Shift Assay (EMSA)

Gel shift assays were performed according to the manufacturer’s recommendations (Promega, Madison, WI, USA). The oligonucleotide sequences for NF-κB were 5′-AGT TGA GGG GAC TTT CCC AGG C-3′. Consensus oligonucleotides were end-labeled using T4 polynucleotide kinase and [^32^P] ATP for 10 min at 37 °C. Briefly, 10 μg nuclear protein were incubated with the labeled probe for 20 min at room temperature. Subsequently, 1 μL of gel loading buffer was added to each reaction and loaded onto a 5% non-denaturing gel and electrophoresis until the dye was three-fourths of the way down the gel. The gel was dried at 80 °C for 1 h and exposed to film overnight at 70 °C. Quantification and the relative density of the protein bands were performed by phosphorimaging (UVP Inc., Upland, CA, USA).

### 4.10. Cell Culture

The RAW 264.7 murine macrophage cell line was obtained from the Korea Cell Line Bank (Seoul, Korea). These cells were grown at 37 °C in Dulbecco’s modified Eagle’s medium (DMEM) medium supplemented with 10% FBS, penicillin (100 units/mL) and streptomycin sulfate (100 μg/mL) in humidified atmosphere of 5% CO_2_. Cells were incubated with TECA at various concentrations (1, 2, or 5 μg/mL) or positive chemicals and then stimulated with LPS 1 μg/mL for the indicated time in figure legends. Various concentrations of TECA dissolved in ethanol were added together with LPS. The final concentration of ethanol used was less than 0.05%. Cells were treated with 0.05% ethanol as vehicle control.

### 4.11. Nitrite Quantification Assay

The NO was determined through the indication of nitrite level in the cell culture media. The RAW264.7 murine macrophages were seeded in 6-well plates (1 × 10^6^ cells/well) with 2 mL of cell culture media and incubated for 24 h. This was followed by discarding the old culture media and replacing them with the new media to maintain the cells. Different concentrations of TECA (1, 2, and 5 μg/mL) were pretreated with the RAW264.7 macrophages. Induction of RAW264.7 macrophages with LPS (1 μg/mL) for all samples was conducted except in control for another 24 h. Then, 100 μL of the collected supernatants was added with 100 μL of Griess reagent (0.1% *N*-1-napthylethylenediamine dihydrochloride (NED), 1% sulphanilamide, and 2.5% phosphoric acid) and incubated in room temperature for 10 min in dark condition. The absorbance was determined by using a microplate reader at 540 nm wavelength. The NO concentration was determined by comparison to the standard curve.

### 4.12. Statistical Analysis

The experiments were conducted in triplicate, and all experiments were repeated at least three times with similar results. All statistical analysis was performed with GraphPad Prism 5 software (Version 5.03; GraphPad software, Inc., San Diego, CA, USA). Group differences were analyzed by one-way ANOVA followed by Tukey’s multiple comparison test. All values are presented as mean ± SD. Significance was set at *p* < 0.05 for all tests.

## 5. Conclusions

In our present study, TECA treatment inhibited activation of NF-κB contributing to the reduced pro-inflammatory cytokine and expression of iNOS and COX-2 in PA-induced allergic dermatitis animal model as well as RAW 264.7 murine macrophage. Therefore, our data suggest that TECA could be a promising agent for AD.

## Figures and Tables

**Table 1:** Composition of titrated extracts of *Centella asiatica*.

Extract	Composition of Extract
Titrated extract of *Centella asiatica* (TECA)	Asiaticoside (40%), Asiatic acid (30%), Madecassic acid (29–30%)

Titrated extract of *Centella asiatica* includes 40% of asiaticoside, 30% of asiatic acid, and 29–30% of madecassic acid.
